# The management and outcome of paediatric splenic injuries in the Netherlands

**DOI:** 10.1186/s13017-021-00353-4

**Published:** 2021-02-27

**Authors:** Maike Grootenhaar, Dominique Lamers, Karin Kamphuis-van Ulzen, Ivo de Blaauw, Edward C. Tan

**Affiliations:** 1 Department of Surgery, Radboud University Medical Centre, P.O. Box 9101, 6500 HB Nijmegen, The Netherlands; 2 Department of Orthopaedic Surgery, Radboud University Medical Centre, P.O. Box 9101, 6500 HB Nijmegen, The Netherlands; 3 Department of Radiology, Radboud University Medical Centre, P.O. Box 9101, 6500 HB Nijmegen, The Netherlands; 4 Department of Paediatric Surgery, Radboud University Medical Centre, P.O. Box 9101, 6500 HB Nijmegen, The Netherlands

**Keywords:** Paediatric, Splenic injury, Trauma, Length of stay

## Abstract

**Background:**

Non-operative management (NOM) is generally accepted as a treatment method of traumatic paediatric splenic rupture. However, considerable variations in management exist. This study analyses local trends in aetiology and management of paediatric splenic injuries and evaluates the implementation of the guidelines proposed by the American Paediatric Surgical Association (APSA) in a level 1 trauma centre.

**Methods:**

The charts of paediatric patients with blunt splenic injury (BSI) who were admitted or transferred to a level 1 trauma centre between 2003 and 2020 were retrospectively assessed. Information pertaining to demographics, mechanism of injury, injury description, associated injuries, intervention and outcomes were analysed and compared to international literature.

**Results:**

There were 130 patients with BSI identified (63.1% male), with a mean age of 11.3 ± 4.0 and a mean Injury Severity Score (ISS) of 21.6 ± 13.7. Bicycle accidents were the most common trauma mechanism (23.1%). Sixty-four percent were multi-trauma patients, 25% received blood transfusions, and 31% were haemodynamically unstable. Mean injury grade was 3.0, with 30% of patients having a high-grade injury. In total, 75% of patients underwent NOM with a 100% efficacy rate. Total splenectomy rate was 6.2%. Four patients died due to brain damage.

Patients with a high-grade BSI (grades IV–V) had a significantly higher ISS and longer bedrest and more often presented with an active blush on computed tomography (CT) scans than patients with a low-grade BSI (grades I–III). Non-operative management was mainly the choice of treatment in both groups (76.6% and 79.5%, respectively). Haemodynamic instability was a predictor for operative management (OM) (*p* = 0.001). Predictors for a longer length of stay (LOS) included concomitant injuries, haemodynamic instability and OM (all *p* < 0.02). Interobserver agreement in the grading of BSI is moderate, with a Cohens Kappa coefficient of 0.493.

**Conclusion:**

Non-operative management has proven to be a realistic management approach in both low- and high-grade splenic injuries. Consideration for operative management should be based on haemodynamic instability. Compared to the anticipated length of bedrest and hospital stay outlined in the APSA guidelines, the Netherlands can reduce the length of bedrest and hospital stay through their non-operative management.

**Level of evidence:**

Therapeutic study, level III

**Supplementary Information:**

The online version contains supplementary material available at 10.1186/s13017-021-00353-4.

## Background

Trauma is the leading cause of death and disability in children. More than 90% of the paediatric trauma admissions worldwide are the result of a blunt mechanism, with 10% of these involving injury to the abdomen and pelvis [1, 2]. The spleen is the most commonly injured intra-abdominal organ in children [2]. Compared to adults, the abdominal organs in children are at a higher risk of organ lesions due to a higher transmission rate of forces through the thinner abdominal wall, larger relative surfaces of the spleen and liver, more flexible ribs and the more horizontal positioning of the diaphragm in children compared to adults [3].

The management of splenic injury in children has evolved since 1968, the year in which Upadhyaya and Simpson introduced non-operative treatment in children with splenic injury [4]. Non-operative management (NOM) has become the standard care for haemodynamically stable patients with blunt splenic injuries without other indications for abdominal surgery. These patients are commonly managed with strict bedrest and close monitoring of vital signs in the hospital. The immediate success of non-operative management at the moment exceeds 95% [5–7]. Bedrest is also associated with minimal risk for long-term complications, such as secondary haemorrhage and reduction of length of stay without increasing the failure rates of NOM [6, 8]. However, bedrest has also been associated with complications such as pneumonia [9] (however, this was not seen in our cohort). Furthermore, it reduces the risk of blood utilisation and avoids increased risk of overwhelming post-splenectomy infection (OPSI) in these patients. Although OPSI has a low incidence of 1–4.5% in the total population (depending on immunologic maturity), it has an exceedingly high mortality rate of 80% [10–12].

Previous studies conducted in Canada and the USA comparing operative and non-operative management showed great variation in management between hospitals and regions [13–15]. Trauma patient volumes in European centres are often considerably lower than in the USA. Therefore, it is questionable whether these results can be translated to centres with lower volumes of patients with blunt splenic injuries. Variation is also observed within non-operative treatments in Europe [16, 17].

There is currently little data investigating the management and outcomes of paediatric spleen trauma in Europe. This study determines if the internationally accepted guidelines first proposed by Stylianos et al. [18] and the American Paediatric Surgical Association (APSA) committee on non-operative management of traumatic spleen ruptures (both isolated and as part of a multi-trauma) are applicable to the Netherlands. Secondarily, we intend to determine how the outcomes of BSI management in the Netherlands compare to those of foreign hospitals.

The current method of grading splenic injuries in the Netherlands is carried out by a radiologist in the acute trauma setting. Assessment is based on computed tomography scans (CT) made upon admission, and the grades are classified according to the American Association for the Surgery of Trauma (AAST) [19]. This study also investigates interobserver variability in the assessment of injury grades between the initial assessment and the prospective reassessment by a paediatric radiologist.

Although that there are various studies related to non-operative methods to paediatric spleen injuries in general, there are only a few discussing the successfulness of NOM in patients with severe splenic injury (grades IV–V) [20]. Hence, this study investigates if NOM is a feasible treatment option for high-grade splenic injuries.

## Methods

### Study design

This is a retrospective single centre cohort study including paediatric patients younger than 18 years with a diagnosis of BSI. Patients were included when admitted or transferred to our level 1 trauma centre with a specialised paediatric surgical department (Radboudumc) between 1 January 2003 and 1 May 2020. The paediatric age group was defined as age < 18 years which conforms the age limit for admittance to paediatric hospitals in the Netherlands. Cases meeting the inclusion criteria were identified through the National Trauma Registration (NTR), giving a total study population of 130 patients.

### Data collection

After acquiring institutional review board approval, patient and injury data were collected from electronic hospital charts. Data pertaining to demographics such as age, gender and admission centre were collected. Clinical data obtained included emergency department vital signs, transfusion requirements, Glasgow Coma Scale (GCS), Injury Severity Score (ISS), haemodynamic status, mechanism of injury, splenic injury grade, and other associated injuries. Furthermore, type of management (NOM or operative management [OM]), duration of stay in the ward and intensive care unit (ICU), overall length of stay (LOS), period of bedrest, morbidity, mortality, and follow-up were collected. The use of the post-splenectomy protocol was also examined [14], where applicable.

Injury Severity Score was obtained from the NTR. Haemodynamic instability was defined as a systolic blood pressure below 100mmHg, a heart rate above 120/min, and the need for transfusion [21]. As these parameters differ by age, assessment of the haemodynamic status from the on-call ER physician and trauma surgeon was used. Injury grades were initially classified within 2 days of admission according to the AAST classification by the on-call radiologist using the CT scans made upon admission. Injury grades were additionally classified prospectively by a paediatric radiologist using the same CT scans in July 2020. Low-grade splenic injuries were classified as grades I–III, while grades IV and V were categorised as high-grade injuries [20, 22]. Non-operative management was defined as bedrest and monitoring of vital signs, including embolisation of the spleen as it is a less invasive procedure. Operative management was defined as splenectomy, spleen-preserving procedures (including partial splenectomy, splenorrhaphy, and laparotomy only), or an intervention to another organ besides the spleen. All data were collected in an electronic database (Castor Electronic Data Capture, released in 2012). The primary outcome of this study is days of bedrest and overall LOS. Secondary outcome measures are duration of stay in the ward and in the ICU as well as mortality.

### Statistical analysis

Statistical analyses were performed using commercially available software (SPSS, version 25.0; licensed by IBM). Descriptive statistics were generated for each variable and compared with current international literature. Continuous variables are described as mean ± standard deviation (SD), whereas categorical variables are reported as frequency and percentage (*n*, %). Comparisons between groups were performed using the independent *t* tests or Mann-Whitney *U* tests for continuous variables and the chi-squared tests and Fisher’s exact tests for categorical variables depending on the variables’ distribution (which was determined with the Kolmogorov-Smirnov *Z* test). Independent risk factors for a longer LOS were determined using a multivariate linear regression model. Independent risk factors for operative management (for both the general population and the high-grade injury group) were identified via a multivariable logistic regression analysis. In order to determine inter-observer agreement in injury grading between the initial assessment (in the acute trauma setting) and the additional second assessment (by a paediatric radiologist), the Cohen’s kappa coefficient was calculated. All *p* values were two-sided and were considered statistically significant if *p* < 0.05.

## Results

### Patient characteristics

From 2003 to 2020, 130 children were admitted with BSI. Sixty-two patients (47.7%) were transferred to our hospital, while 68 (52.3%) arrived from scene. Blunt splenic injury most often occurred during spring (34.6%). The study population had a median age of 12 years [range 3–17], of whom the majority were male (63.1%). As presented in Table 1, falls from height were the most common cause of BSI (16.9%), followed by bicycle-vehicle collisions (16.2%) and individual bicycle injuries (15.4%). Those trauma mechanisms preceding BSI were categorised as high-energetic trauma (HET) or low-energetic trauma (LET) in 68.5% and 31.5% of cases, respectively. The LET group was mostly represented by individual bicycle accidents (26.8%). Additional patient demographics of the entire cohort are listed in Table 1.

**Table 1:** Characteristics of study population

Total study population (*N* = 130)	
*Age in years, median [range]*	12 [3–17]
*Age in years, N (%)*	
< 6 years	13 (10.0)
6–10 years	45 (34.6)
> 10 years	72 (55.4)
*Sex, N (%)*	
Male	82 (63.1)
Female	48 (36.9)
*Trauma mechanism, N (%)*	
Accidents involving a motorised vehicle	59 (45.4)
Vehicle-vehicle	14 (10.8)
Bicycle-vehicle	21 (16.2)
Pedestrian-vehicle	10 (7.7)
Single vehicle	14 (10.8)
Bicycle accidents	20 (15.4)
Falls	30 (23.1)
Falls from height (> 1 m)	22 (16.9)
Falls on same level (<1m)	8 (6.2)
Stump object in abdomen	21 (16.2)
Kick from a horse	11 (8.5)
Sports/bumping into obstacles	10 (7.7)
*ISS, mean ± SD*	21.6 ± 13.7
*Splenic injury grade, N (%)*	
Grade I	11 (8.5)
Grade II	23 (17.7)
Grade III	43 (33.1)
Grade IV	35 (26.9)
Grade V	4 (3.1)
Undetermined	14 (10.8)
*Presence of active blush on CT, N (%)*	21 (16.2)
*Isolated splenic injury, N (%)*	47 (36.2)

*Abbreviations: CT* computerised tomography, *ISS* Injury Severity Score, *SD* standard deviation

### Injury characteristics

Nearly 64% of patients were multi-trauma patients with concomitant injuries. Multi-trauma patients needed to be transfused more often (30.1% vs 14.9% in the isolated injury group; *p* = 0.037), had a higher ISS (26.7 ± 14.3 vs 12.2 ± 4.9; *p* < 0.001), and were more often haemodynamically unstable (37.3% vs 19.1%; *p* = 0.031). Clinical injury data including the distribution of concomitant injuries are presented in Supplementary Table 1. The mean ISS of BSI patients was 21.6 ± 13.7. In 20.3% of cases, GCS was lower than 8, indicating a severely injured neurological state. Mean haemoglobin (Hb) concentration during admission was 7.2 ± 1.1 mmol/l. Of all patients, 30.8% were haemodynamically unstable.

They had a significantly higher ISS (32.2 ± 14.6 vs 17.5 ± 11.0 in the stable group; *p* < 0.001) and a lower serum Hb concentration on admission (6.2 ± 1.1 vs 7.5 ± 0.9; *p* < 0.001), leading to more blood transfusions (77.5% vs 1.1%; *p* < 0.001), and they more often showed an active blush on CT scan (22.5% vs 10.0%; *p* = 0.006). Fifty-nine percent of patients had a low-grade splenic injury (grades I–III) and 30.5% of patients had a high-grade splenic injury (grades IV–V) based on their CT scan on admission, with a mean splenic injury grade of 3.0 (± 1.0). The distribution in splenic injury grade is presented in Table 1. An active blush was present in 16.2% of patients. Prior to a CT scan, an abdominal ultrasound was performed for 81.5% of patients to detect abdominal trauma or free fluid.

### Management

The majority of patients with BSI were treated non-operatively (74.6%), including embolisation of the spleen due to haemodynamic instability and the presence of an arterial blush. Of the patients undergoing OM, 24.2% had a splenectomy, 18.2% had a spleen-preserving procedure, and 57.6% experienced an intervention on another organ besides the spleen. Splenectomy rate for the entire cohort was 6.2%. The main reasons for opting for OM were haemodynamic instability (71.4%), presence of arterial blush (7.1%), and persistence of abdominal pain (7.1%). Age and ISS were higher among the OM group than in the NOM group (12.9 ± 3.9 vs 10.8 ± 4.0; *p* = 0.009 and 33.9 ± 13.5 vs 17.5 ± 11.2; *p* < 0.001, respectively). Moreover, patients in the OM group more often needed transfusion (45.5% vs 17.5%; *p* < 0.001) and were haemodynamically unstable more frequently (66.7% vs 18.6%; *p* < 0.001), and all were multi-trauma patients due to a HET (*p* < 0.001). Multivariable logistic regression analysis shows that being haemodynamically unstable is an independent predictor for OM (odds ratio [OR] 0.151 [95% confidence interval (CI) 0.05–0.45]; *p* = 0.001).

The post-splenectomy protocol was followed by 75.0% of patients who underwent splenectomy and 40.0% of those who underwent embolisation. They all received pneumococcal vaccinations and in some cases additional influenza and meningococcal vaccinations. Eighty-nine percent of our cohort received pain medication, consisting most often of paracetamol (84.9%) or opioids (64.2%). Follow-up after BSI mostly consisted of clinical check-ups (25.7%) that were sometimes combined with secondary ultrasound of the spleen (13.3%). Follow-up time varied from 1 to 8 weeks, with an average of 3 weeks. After discharge, doctors advised a return to activities within 7 weeks in 77.0% of cases. This was extended to > 7 weeks for multi-trauma or operated patients.

### Treatment outcomes

The main treatment outcomes of BSI are displayed in Table 2. One-hundred-and-three patients (79.2%) were admitted to the ICU during admission. After bedrest, initialisation of mobilisation/ambulation was delayed with a mean of 5.3 ± 3.5 days. The duration of stay in the ward, duration of stay in the ICU, and LOS were significantly higher (*p* < 0.01) in patients that were either haemodynamically unstable, multi-trauma patients or managed operatively. The amount of bedrest was significantly higher (*p* < 0.03) in both unstable patients and when undergoing OM.

**Table 2:** Treatment outcomes for blunt splenic injuries

Treatment outcomes: Mean ± SD [range]	Total	High-grade	Low-grade
Length of ward stay (in days)	6.8 ± 5.9 [0–24]	6.5 ± 4.8 [0–24]	6.6 ± 6.4 [0–24]
Length of ICU stay (in days)	3.5 ± 5.6 [0–30]	3.8 ± 5.5 [0–30]	3.5 ± 6.0 [0–29]
LOS (in days)	10.2 ± 9.0 [1–43]	10.1 ± 8.0 [1–39]	10.2 ± 9.7 [2–43]
Bedrest (in days)	5.0 ± 3.1 [0–16]	5.5 ± 2.6 [2–16]	4.8 ± 3.3 [0–14]
Splenic complications	No splenic complications		
In-hospital mortality (% of all patients)	3.1	2.6	1.3

*Abbreviations: ICU* intensive care unit, *LOS* length of stay, *SD* standard deviation

According to the multivariate linear regression analysis, independent predictors for an extended LOS are OM (unstandardised regression coefficient [*β*] 6.98 [95% CI 2.98–10.98]; *p* = 0.001), unstable haemodynamics ([*β*] 4.97 [95% CI 1.46–8.48]; *p* = 0.006) and presence of concomitant injuries ([*β*] 4.80 [95% CI 8.59–1.01]; *p* = 0.014). These predictors explain a significant part of the variance in LOS (adjusted *R*-squared = 0.311; *F*(6,109) = 9.664; *p* < 0.001). Patients had a longer LOS of 7.0 days when managed operatively, 5.0 days when they were haemodynamically unstable and 4.8 days when they were multi-trauma patients.

Although no complications related to splenic injury occurred, 20.0% endured complications from another origin. Most often these were neurological or psychiatric complications (34.6% and 50.0%, respectively) due to multi-trauma. Four patients (3.1%) died within 10 days of admission due to non-survivable brain damage. They were multi-trauma patients with an ISS exceeding 50.

A comparison of the clinical data and outcomes of management between low-grade and high-grade splenic injuries is depicted in Table 3.

**Table 3:** Comparison of characteristics: low-grade vs high-grade splenic injuries

	Low-grade (*N* = 77)	High-grade (*N*=39)	*P* value
Age (in years)	11.0 ± 4.0	11.6 ± 4.0	0.359^c^
Male (%)	62.3	66.7	0.647^a^
Serum Hb (in mmol/l)	7.5 ± 1.0	6.6 ± 1.2	**0.005**^**c**^
Need of blood transfusion (%)	18.2	35.9	**0.047**^**a**^
ISS (in points)	19.4	24.4	**0.022**^**c**^
Trauma mechanism that occurred most (%)	Bicycle vs vehicle accident (19.5)	Bicycle accident (20.5)	0.960^b^
LET (%)	32.5	30.8	0.853^a^
HET (%)	67.5	69.2	0.853^a^
Presence of active blush (%)	9.1	30.8	**0.002**^**a**^
Haemodynamic stability			
Stable (%)	76.6	64.1	0.154^a^
Unstable (%)	23.4	35.9	
Isolated splenic injury (%)	32.5	48.7	0.088^a^
Management			
NOM (%)	76.6	79.5	0.727^a^
OM (%)	23.4	20.5	0.727^a^
Embolisation (%)	0.0	12.8	**0.004**^**b**^
Splenectomy (%)	2.6	7.7	0.333^b^
Stay in ward (in days)	6.6 ± 6.4	6.5 ± 4.8	0.134^c^
Stay in ICU (in days)	3.5 ± 6.0	3.8 ± 5.5	0.160^c^
LOS (in days)	10.2 ± 9.7	10.1 ± 8.0	0.058^c^
Bedrest (in days)	4.8 ± 3.3	5.5 ± 2.6	**0.004**^**c**^
Mortality (%)	1.3	2.6	0.561^b^

Bold parameters are significant (as *P* value < 0.05)

*Abbreviations*: *Hb* haemoglobin, *HET* high-energetic trauma, *ICU* intensive care unit, *ISS* Injury Severity Score, *LET* low-energetic trauma, *LOS* length of stay, *NOM* non-operative management, *OM* operative management

^a^Chi-squared test

^b^Fisher’s exact test

^c^Mann-Whitney *U* test

### Differences between OM and NOM in patients with a high-grade BSI

Of the patients with a high-grade splenic injury (*n* = 39), 79.5% underwent NOM. Five of these patients were treated with embolisation, due to haemodynamic instability or the presence of an active blush on their CT scan. Of those managed operatively (20.5%), three had a splenectomy (37.5%), one underwent a laparotomy (12.5%) and one had a spleen-preserving procedure (12.5%).

A comparison of the clinical data and outcomes of management for the NOM and OM groups for patients with a high-grade splenic injury is depicted in Table 4. Patients who were treated operatively were significantly older (15.6 ± 1.8 vs 10.6 ± 3.7; *p* < 0.001), had a higher ISS (43.7 ± 7.1 vs 20.2 ± 7.9; *p* < 0.001) and had an extended LOS (16.1 ± 10.9 vs 8.5 ± 6.4; *p* = 0.013). Similarly, operatively treated patients were all multi-trauma patients due to a HET (*p* = 0.003 and *p* = 0.042, respectively). The multivariable logistic regression analysis shows that age is an independent predictor for OM (OR 0.855 [95% CI 0.75–0.97]; *p* = 0.015).

**Table 4:** Comparison of characteristics: NOM vs OM in patients with high-grade splenic injury

	Non-operative (*N* = 31)	Operative (*N* = 8)	*P* value
Age (in years)	10.6 ± 3.7	15.6 ± 1.8	**0.000**^**b**^
Male (%)	64.5	75.0	0.694^a^
Serum Hb (in mmol/l)	6.7 ± 1.2	6.3 ± 1.6	0.223^c^
Need of blood transfusion (%)	32.3	50.0	0.424^a^
ISS (in points)	20.2 ± 7.9	43.7 ± 7.1	**0.000**^**b**^
Trauma mechanism that occurred most (%)	Bicycle accident (22.6)	Vehicle vs vehicle accident (37.5)	0.223^a^
LET (%)	38.7	0.0	**0.042**^**a**^
HET (%)	61.3	100.0	
Presence of active blush (%)	29.6	50.0	0.402^a^
Haemodynamic stability			
Stable (%)	71.0	37.5	0.109^a^
Unstable (%)	29.0	62.5	0.109^a^
Isolated splenic injury (%)	61.3	0.0	**0.003**^**a**^
Stay in ward (in days)	5.8 ± 3.8	8.9 ± 7.4	0.150^b^
Stay in ICU (in days)	2.9 ± 3.4	7.3 ± 10.0	0.184^b^
LOS (in days)	8.5 ± 6.4	16.1 ± 10.9	**0.013**^**b**^
Bedrest (in days)	5.4 ± 2.3	6.5 ± 4.4	0.932^b^
Mortality (%)	0.0	12.5	0.205^a^

Bold parameters are significant (as *P* value < 0.05)

*Abbreviations*: *Hb* haemoglobin, *HET* high-energetic trauma, *ICU* intensive care unit, *ISS* Injury Severity Score, *LET* low-energetic trauma, *LOS* length of stay

^a^Fisher’s exact test

^b^Mann-Whitney *U* test

^c^Independent sample *t* test

### Interobserver variability

The difference in injury grades, as determined by the radiologist in the acute trauma setting and reassessed by a paediatric radiologist, is presented in Table 5. Blunt splenic injuries tend to be graded higher in the acute trauma setting, with a mean injury grade of 3.0 ± 1.10, as compared to reassessment by a paediatric radiologist, with a mean injury grade of 2.7 ± 1.37. However, this difference was not significant (*p* = 0 .519). The Cohens Kappa coefficient was 0.493, indicating that interobserver agreement is moderate.

**Table 5:** Interobserver variability: initial vs rescored injury grade

	Grade	Rescored^b^						Total
		0	I	II	III	IV	V	
Initial^a^	**0**	0	0	0	0	0	0	0
	**I**	2	6	2	0	0	0	10
	**II**	0	1	15	3	0	0	19
	**III**	1	0	7	26	7	0	41
	**IV**	0	0	1	13	17	1	32
	**V**	0	0	0	0	1	3	4
Total		3	7	25	42	25	4	106

^a^Initial grading (based on the AAST score) was performed in the acute trauma setting by the radiologists on call

^b^Prospective rescoring of injury grades was done by Karin Kamphuis-van Ulzen, a paediatric radiologist at Radboudumc Nijmegen

Furthermore, presence of an active injury on a CT scan in the acute trauma setting was detected significantly more often than during reassessment (16.2% vs 10.0%; *p* < 0.001).

## Discussion

This study shows that non-operative management is still the best management option for patients with splenic injuries that are haemodynamically stable. However, operative treatment may be necessary, especially in patients who are haemodynamically unstable, which is usually apparent on admission or within 12 h of injury [18, 23]. In accordance with the bedrest and hospitalisation periods deemed safe by the APSA guidelines, shorter periods of bedrest can be applied in our hospital as per the results of this study.

### Management

This study shows no crossover between initial NOM to OM regardless of injury grade, thus giving a 100% success rate for NOM for both low-grade and high-grade splenic injuries; this is better than international splenic salvage rates reaching a maximum of 99% for isolated injuries in the last 30 years [24]. Non-operative treatment has proven to be associated with minimal risk of short- and long-term complications such as haemorrhages, abscesses, pseudo-aneurysms and (pseudo-) cysts. In our cohort, no splenic complications occurred during the follow-up time of a maximum of 8 weeks. Managing a paediatric patient non-operatively spares the additional risk of overwhelming infections associated with asplenic patients and gives them better quality of life measurements compared to those who undergo splenectomy [12, 25–27].

The success of NOM increases with splenic artery embolisation even in high-grade injuries in 3.8% of cases (all high-grade BSI). Recent research in the paediatric population shows that embolisation can be a viable alternative to splenectomy when non-operative treatment fails [28]. It has been demonstrated that immune function after splenic artery embolisation is similar to patients who have not been embolised [28, 29]. Splenic artery embolisation has been possible since 2009 in our hospital; as such this treatment option was not accessible to a portion of our cohort.

A relatively low percentage of patients who underwent a splenectomy (6.2%) was found in relation to comparable international studies [1, 13, 20, 30–38] (Supplementary Table 3) and studies performed in the Netherlands, in which 17.4% and 21.3% of patients underwent a splenectomy, respectively [16, 39]. This study supports that unstable haemodynamics is an independent risk factor for splenectomy in children [5], possibly in combination with the presence of an active blush on the CT scan. Post-splenectomy protocol has also altered in recent years, and now post-operative vaccination is routinely implemented.

### APSA guidelines

The actual duration of stay in the ICU and total length of stay for isolated splenic injuries treated with NOM compared to the APSA guidelines are presented in Supplementary Table 2. As shown in the table, ICU stay and LOS in our hospital are almost equal to the guidelines. Even though the current protocol has been implemented since 2006, its implementation is still an ongoing process. This study observed a mean stay at the hospital of 5.6 ± 2.1 days in isolated splenic injuries and 7.7 ± 6.8 days in conservatively treated patients. Related studies in the Netherlands show higher lengths for hospital stay (11.9 ± 5.2 days and 7–16 days after 2000) [16, 39]. This study shows that the length of stay is shorter than in other related studies in the Netherlands. Thus, our results suggest that the APSA guidelines are safe and applicable to the Dutch context and that our hospital can apply a shorter period of bedrest and hospitalisation for children with splenic injuries.

### Grading splenic injury

This study addresses an unstudied practice: the prospective assessment of the injury grade of splenic injuries based on CT scans. Review of current literature on the grading of splenic injury using CT scans and predicting the optimal management of BSI shows differing opinions regarding whether injury grade is a determinant of splenectomy [40–42]. After rescoring all available CT scans, moderate agreement was found between the initial radiologist in the acute trauma setting and the paediatric-radiologist in the research setting. In other words, slight overscoring of splenic injuries occurred in the acute trauma setting. One could argue that in the acute trauma setting a radiologist is more prone to ‘upscale’ the grading as there is sometimes limited time and the radiologist is not specialised in paediatric trauma. However, no evidence was found to support this theory. It is important to do further research on how to improve the correctness of agreement on the scoring of splenic injuries because different scores could possibly change the management decision and therefore the length of hospitalisation [43]. For this reason, we recommend a specialised paediatric radiologist to be available for the emergency department at all times to reassess the CT scans during admission or to help assess the CT scan before management is determined.

### Dutch-specific aetiology

This study describes a Dutch-specific aetiology in splenic injuries. Studies performed in the Netherlands showed data supporting the contribution of motor vehicle accidents (45.4%), falls (23.1%) and bicycle accidents (15.4%) to splenic injury [16]. An interesting difference between this study and studies performed internationally is that a higher percentage of bicycle accidents occurred as the trauma mechanism for BSI in the Netherlands (see Supplementary Table 3) [31, 35–38]. This difference in aetiology can be explained by the fact that in the Netherlands (and in the UK as well), the bicycle is an important means of transportation for adults and children alike. Similar findings on aetiology were found in paediatric pancreatic trauma [44].

### Limitations

The present study has several limitations. First, this study is susceptible to the limitations that come with all retrospective studies using national administrative databases, namely missing data due to inaccurate recordkeeping. As our study only uses the electronic hospital charts from our hospital, we could not obtain long-term patient outcomes and complications after patients were transferred. In the future, meticulous record keeping can result in more complete studies in this particular field. Nevertheless, our clinic is representative of the Netherlands as a paediatric trauma clinic and our management is coordinated with the Dutch Association for Paediatric Surgery. Our research demonstrates that non-operative management can be chosen regardless of injury grade. In addition, we identified independent predictors for operative management and a longer length of stay.

This study covers a long time period for admissions (stretching over 17 years) in which much has changed in the management of paediatric splenic injuries. In the earlier years, there was an inclination towards OM of high-grade injuries (28.6% before the implementation of embolisation in 2009 vs 18.7% after implementation; *p =* 0.617). Presently, a paradigm shift can be observed in that patients with a high-grade splenic injury who are haemodynamically stable are sometimes treated conservatively. The APSA guidelines have clear recommendations concerning splenic injuries of grades I to IV. However, recent literature about therapy on grade V splenic injuries is rare. This study supports the strategy to initially choose NOM in all high-grade injuries as the majority of patients (almost 80.0%) are successfully managed conservatively, with a 100% accuracy rate. Indeed, this percentage is an underestimation given that 37.5% of the patients in the high-grade group who were managed operatively needed surgery for an injury other than their splenic rupture. Even though NOM might be possible as the initial treatment in some cases, the decision between NOM and OM depends on careful risk-benefit analysis for each patient, particularly taking into account haemodynamic status and the expertise of the surgeon and the multidisciplinary hospital team. More recent evidence suggests that for higher grade injuries angioembolisation may also be suitable as an adjunct to NOM if the patient becomes haemodynamically unstable [45–49]. In our hospital, this technique has been available for children since 2009.

A striking observation of differences in age limit occurs when looking at Supplementary Table 3. In all studies, only paediatric patients with BSI were included. However, a diverse variation in the definition of ‘paediatric’ is used, ranging from < 16 years to < 22 years of age. Hence, comparisons between studies should be performed cautiously.

Moreover, the study conducted is a single-institution study, which creates a small sample size, and therefore, the results might not be generalisable to the Dutch population. To allow better comparison with international study outcomes, a nationwide study with all level I trauma centres in the Netherlands would be the next logical step in future studies. Though sample size is relatively small, the scarce number of publications considering NOM in high-grade splenic injuries and the proven success of NOM enhances the significance of our study. The treatment protocol used in our hospital is in accordance with national paediatric trauma guidelines.

## Conclusion

To our knowledge, studies documenting the aetiology, management and outcome of BSI in children in Europe and the Netherlands are scarce. In paediatric blunt splenic trauma, NOM is the treatment of choice in haemodynamically stable children regardless of injury grade. Few complications are observed following this method of management. Our advice is to follow the APSA guidelines more effectively by reducing hospital admissions. Initial grading of splenic injuries is initially overestimated, though the decision in management of BSI still depends on haemodynamic status, meaning that overscoring of injury grade does not influence management.

## Supplementary Information

**Additional file 1: Supplementary Table 1**: Distribution of concomitant injuries

**Additional file 2: Supplementary Table 2**: Proposed APSA guidelines vs actual treatment

**Additional file 3: Supplementary Table 3**: International Comparable Studies

## Data Availability

The datasets used and/or analysed during the current study are available from the corresponding author on reasonable request.

## Abbreviations

AAST: American Association for the Surgery of Trauma
APSA: American Paediatric Surgical Association
β: Unstandardised regression coefficient
BSI: Blunt splenic injury
CI: Confidence interval
CT: Computed tomography
NOM: Non-operative management
GCS: Glasgow Coma Scale
Hb: Haemoglobin
HET: High-energetic trauma
ICU: Intensive care unit
ISS: Injury Severity Score
LET: Low-energetic trauma
LOS: Length of stay
*N*: Number
NTR: National Trauma Registration
OM: Operative management
OPSI: Overwhelming post-splenectomy infection
OR: Odds ratio
SD: Standard deviation
SPA: Splenic pseudo-aneurysms
US: United States
