# Supplementary material for: The management and outcome of paediatric splenic injuries in the Netherlands
Source: World J Emerg Surg. 2021 Feb 27;16:8. doi: 10.1186/s13017-021-00353-4 (PMC7913258; doi:10.1186/s13017-021-00353-4)
Supplement: Supplementary file 1 — Additional file 1: Supplementary Table 1: Distribution of concomitant injuries [file 13017_2021_353_MOESM1_ESM.docx]

Additional file 1

**Supplementary Table 1: Distribution of concomitant injuries**

| Concomitant injury | N (%) |
| --- | --- |
| No concomitant injury (isolated splenic injury) | 47 (36.2) |
| Fractures | 49 (59.0) |
| Thoracic wall / pulmonary | 44 (53.0) |
| Liver | 20 (24.1) |
| Pancreas | 7 (8.4) |
| Kidney / adrenal | 30 (36.1) |
| Gastrointestinal tract | 9 (10.8) |
| Craniocerebral | 37 (44.6) |
| Soft tissue | 25 (30.1) |
| Other | 8 (9.6) |
