# Supplementary material for: The management and outcome of paediatric splenic injuries in the Netherlands
Source: World J Emerg Surg. 2021 Feb 27;16:8. doi: 10.1186/s13017-021-00353-4 (PMC7913258; doi:10.1186/s13017-021-00353-4)
Supplement: Supplementary file 2 — Additional file 2: Supplementary Table 2: Proposed APSA guidelines vs actual treatment [file 13017_2021_353_MOESM2_ESM.docx]

Additional file 2

**Supplementary Table 2: Proposed APSA guidelines vs actual treatment**

|  | CT-grade | |
| --- | --- | --- |
|  | **Low-grade (I-III)** | **High-grade (IV-V)** |
| ICU stay^a^ (days) | None^a^ | 1^a^ |
| Actual treatment^b^ (days ± SD) | 1.1 ± 0.8 | 2.0 ± 1.5 |
| LOS^a^ (days) | 3^a^ | 5^a^ |
| Actual treatment^c^ (days ± SD) | 4.7 ± 2.1 | 6.5 ± 1.6 |

^a^Proposed guidelines; ^b^Actual ICU stay in mean days ± SD; ^c^Actual LOS in mean days ± SD. *Abbreviations: CT* Computerized Tomography, *ICU* Intensive Care Unit, *LOS* Length Of Stay, *SD* Standard Deviation.
